# RELIGIOUS ATTENDANCE AND SLEEP DISTURBANCE IN OLDER MEXICAN AMERICANS

**DOI:** 10.1093/geroni/igz038.1939

**Published:** 2019-11-08

**Authors:** Terrence D Hill, Christopher Ellison, Lauren Hale

**Affiliations:** 1 University of Arizona, Tucson, Arizona, United States; 2 University of Texas at San Antonio, San Antonio, Texas, United States; 3 Stony Brook University, Stony Brook, New York, United States

## Abstract

Although numerous studies have shown that religious involvement is associated with better health across the life course, researchers have virtually ignored possible links between religious involvement and sleep-related outcomes. Building on previous work, we tested whether religious attendance was inversely associated with sleep disturbance among older Mexican Americans. We also assessed whether depressive symptoms could mediate or explain any of the inverse association between religious attendance and sleep disturbance. Relevant hypotheses were tested using ordinary least squares regression and conditional process mediation analysis of cross-sectional data collected from the original cohort of the Hispanic Established Population for the Epidemiologic Study of the Elderly (H-EPESE). The baseline H-EPESE (1993-1994) included a probability sample of 3,050 Mexican Americans ages 65 and older. Due to missing data on our focal variables, our final analytic sample included 2,323 respondents. Regression models show that religious attendance is inversely associated with depressive symptoms and sleep disturbance, even with adjustments for smoking, drinking, body mass, chronic disease, mobility, marital status, living arrangements, family engagement, secular group participation, social support, age, gender, immigrant status, language proficiency, education, household income, and religious affiliation. Mediation analyses also indicate that depressive symptoms fully mediate the association between religious attendance and sleep disturbance. These findings contribute to previous work by showing that regular religious attendance may protect against sleep disturbance by promoting mental health in an understudied population of older Mexican Americans. The importance of religious involvement is supported by the fact that secular group participation was unrelated to sleep disturbance.

